# Immune tolerance to an intestine-adapted bacteria, *Chryseobacterium* sp., injected into the hemocoel of *Protaetia brevitarsis seulensis*

**DOI:** 10.1038/srep31722

**Published:** 2016-08-17

**Authors:** Jiae Lee, Sejung Hwang, Saeyoull Cho

**Affiliations:** 1 Department of Applied Biology, College of Agriculture and Life Science, Environment Friendly Agriculture Center, Kangwon National University, Chuncheon, Republic of Korea

## Abstract

To explore the interaction of gut microbes and the host immune system, bacteria were isolated from the gut of *Protaetia brevitarsis seulensis* larvae. *Chryseobacterium* sp., *Bacillus subtilis, Arthrobacter arilaitensis, Bacillus amyloliquefaciens, Bacillus megaterium*, and *Lysinibacillus xylanilyticus* were cultured *in vitro*, identified, and injected in the hemocoel of *P. brevitarsis seulensis* larvae, respectively. There were no significant changes in phagocytosis-associated lysosomal formation or pathogen-related autophagosome in immune cells (granulocytes) from *Chryseobacterium* sp.-challenged larvae. Next, we examined changes in the transcription of innate immune genes such as peptidoglycan recognition proteins and antimicrobial peptides following infection with *Chryseobacterium* sp. *PGRP-1* and *-2* transcripts, which may be associated with melanization generated by prophenoloxidase (PPO), were either highly or moderately expressed at 24 h post-infection with *Chryseobacterium* sp. However, *PGRP-SC2* transcripts, which code for bactericidal amidases, were expressed at low levels. With respect to antimicrobial peptides, only coleoptericin was moderately expressed in *Chryseobacterium* sp.-challenged larvae, suggesting maintenance of an optimum number of *Chryseobacterium* sp. All examined genes were expressed at significantly higher levels in larvae challenged with a pathogenic bacterium. Our data demonstrated that gut-inhabiting bacteria, the *Chryseobacterium* sp., induced a weaker immune response than other pathogenic bacteria, *E. coli* K12.

The insect immune system is composed of both a cellular arm and a humoral arm^1^. The cellular immune system mainly comprises hemocytes (phagocytes), which phagocytose, encapsulate, and nodulate pathogenic microorganisms^2^. Among the several types of insect hemocyte, granulocytes are the most abundant in mosquitoes and specifically contribute toward immune response^3^. We previously showed that the granulocytes in *Protaetia brevitarsis seulensis* (Coleoptera: Cetoniidae) larvae play a pivotal role in cellular immune responses, and that they perform specific functions, including autophagy-related phagocytosis and nodulation^4^. Apart from granulocytes, plasmatocytes are also a major professional immune cell in many insects, including flies^4^,^5^. Insect phagocytes (usually, granulocytes and plasmatocytes) consume foreign cells and form intracellular phagosomes, which subsequently fuse with endosomes and, finally, with lysosomes, leading to degradation of the foreign material^4^,^6^. Recent studies report that autophagy is intimately linked to innate or adaptive immune effector functions by facilitating pathogen detection and mediating pathogen clearance by phagocytes^4^,^7^,^8^,^9^,^10^. In addition, peptidoglycan recognition proteins (PGRPs) activate phenoloxidase (PO) in insect hemocytes and activated PO oxidizes phenolic molecules to produce melanin around invading pathogens and wounds^11^. As previously described^12^, phenoloxidase activity in the granulocytes of *P. brevitarsis seulensis* larvae was detected and also shown to be an important component of the cellular immune reaction by its ability to induce insect hemolymph melanization in various insects^11^,^13^.

The insect humoral immune system comprises secreted antimicrobial peptides (AMPs). AMPs were originally identified in a soil bacterium, *Bacillus brevis*, and are bactericidal. Since then, over 2,000 AMPs have been identified and characterized in various organisms, including many insects^14^. *Drosophila melanogaster* harbors at least eight classes of AMPs, including lysozyme, defensins, cecropins, drosocin, attacins, diptericin, Maturated Pro-domain of Attain C (MPAC), drosomycin, and metchnikowin, all of which are synthesized by the fat body in response to infection and then secreted into the hemolymph^15^. In insects, these AMPs are generally produced by activation of two major signaling transduction pathways, one activated by fungi and Gram-positive bacteria, and the other by the majority of Gram-negative bacteria and some Gram-positive bacteria. Many signaling molecules, including peptidoglycan recognition proteins (PGRPs), Gram-negative binding proteins (GNBPs), and several proteases, are involved in these two pathways. In addition, other genes, such as those encoding β-1,3-glucan recognition protein (βGRP), scavenger receptor B (SCRB), C-type lectins, hemolin, and integrins, are slightly, moderately, or highly expressed in fat bodies in response to microbe-associated molecular recognition patterns (MAMPs) (e.g., microbial peptidoglycan, lipopolysaccharides, β-glucans, lipoproteins, CpG dinucleotides, or flagellin)^16^,^17^,^18^,^19^,^20^,^21^,^22^. Therefore, changes in expression of host immune genes are determined by virulence factors produced by various bacteria.

Generally, bacteria are highly adaptable to a variety of habitats, including internal organs, and exhibit a large variety of phenotypes ranging from symbiotic to pathogenic^23^,^24^. The general pattern and composition of gut-inhabiting bacteria in diverse insect orders has been described by Yun *et al*.^25^, who reported that the microbiota within the gut of various insects was mainly dominated by *Proteobacteria, Firmicutes, Bacteroidetes, Actinobacteria, Tenericutes*, and unclassified bacteria^25^. *D. melanogaster* has also a moderately complex gut microbiota comprising 5–20 species^26^. How then do gut-inhabiting bacteria cope with the host immune system? The systemic immune responses related to apoptosis, endosome formation, and various AMPs would be less induced in a symbiotic insect^27^. The gut epithelium also play a crucial role as immune receptors which acts as a “selectivity amplifier” to control or maintain beneficial bacteria with only small amounts of weakly selective secretions in human and insect^28^,^29^. Furthermore, germ-free mice show impaired immunological development and are generally more susceptible to infectious with bacterial and viral agents^30^,^31^. Therefore, the gut microbiota are essential for development and maturation of the host immune system^32^. However, it is difficult to study the immunological relationship between single bacterial taxa in the gut and the insect despite the already relative simple composition if the gut microbiota^20^.

We have recently demonstrated that *P. brevitarsis seulensis* larvae is a suitable model for examining insect immune system because they have a well-developed cellular and humoral defense system^4^,^33^. This may be because they live on the ground for over 3 months during the larval stage, where they frequently encounter pathogenic bacteria, fungi, viruses, and parasitoids. In addition, at least 0.5 ml of hemolymph was available from a last instar larva, which was enough to examine cellular immune responses. With the goal of understanding the interaction of immune system and gut microbiota, we first isolated, *in vitro*-cultured, and identified gut-inhabiting bacteria of *P. brevitarsis seulensis* larvae. We then examined host cellular and humoral immune responses to infection by these cultured gut bacteria. Various cellular immunological activities (phagocytosis-related lysosome formation and pathogen-related autophagosome) were examined by microscopy and fluorescence-activated cell sorting. In addition, we examined expression of *PGRP* and *AMP* genes against infection of gut-inhabiting bacteria. These experiments allowed us to show the ability of a gut-inhabiting bacterium to induce a weaker immune response than other pathogenic bacteria, suggesting the existence of a mechanism allowing immune tolerance of this gut symbiont.

## Results

### Isolation of gut bacteria and analysis of the host cellular immune response against them

**Figure 1:**
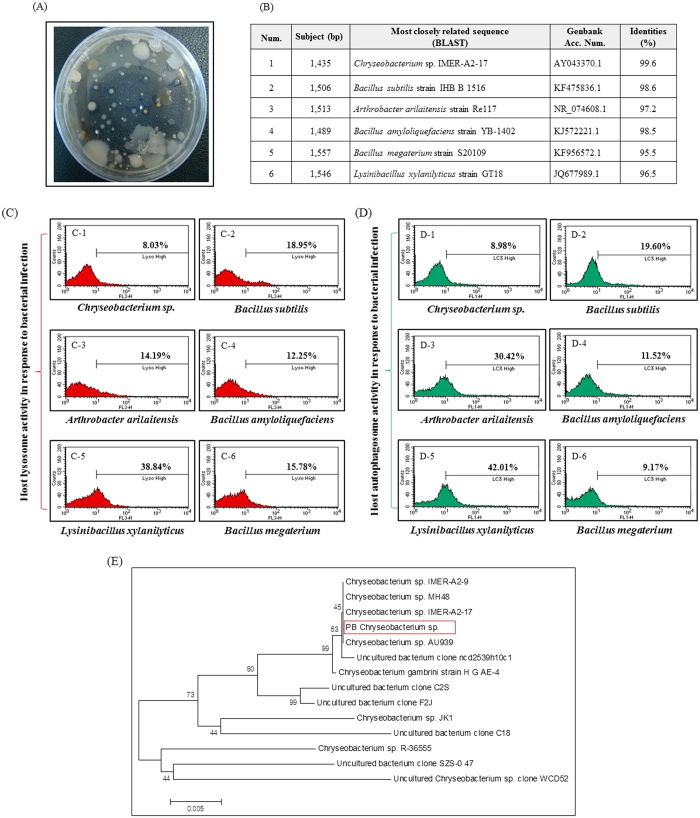
Identification and phylogenetic analysis of *Chryseobacterium* sp. and the host immune responses to the six isolated gut bacteria. (**A**) Bacteria isolated from the gut of *Protaetia brevitarsis seulensis* were cultured on agar plates. Bacterial colonies exhibited different morphologies and growth rates. (**B**) Six bacterial colonies were randomly chosen and the bacterial small subunit ribosomal RNA (16S rRNA) gene was fully sequenced. A BLAST search of the GenBank database revealed that the 16S rRNA gene from all six colonies was >95% identical. The round, yellowish colony was identified as *Chryseobacterium* sp. (**C**) Flow cytometry analysis of LysoTracker Red staining at 12 h post-infection. (**D**) Flow cytometry analysis of green fluroscent-LC3 staining at 12 h post-infection. A low percentage of hemocytes from larvae injected with *Chryseobacterium* sp. were stained with LysoTracker Red (8.03% indicated by red color) and green fluorescent LC3 (8.98% indicated by green color). C-1 and D-1: injection with *Chryseobacterium* sp. (KX371567). C-2 and D-2: injection with *Bacillus subtilis* (KX369580). C-3 and D-3: injection with *Arthrobacter arilaitensis* (KX369581). C-4 and D-4: injection with *Bacillus amyloliquefaciens* (KX369577). C-5 and D-5: injection with *Lysinibacillus xylanilyticus* (KX371346). C-6 and D-6: injection with *Bacillus megaterium* (KX369578). (**E**) The phylogenetic tree showed that *Chryseobacterium* sp. (highlighted in a red box) was closely related to *Chryseobacterium* sp. IMER-A2-17. Species are referenced by strain number and GenBank accession number. Tree building was performed using the neighbor-joining method and fastDNAml^68^. Scale Bar, 0.005 substitutions per base position.

To isolate gut-inhabiting bacteria, we surface-sterilized ten fourth-instar larvae, and dissected and homogenized the alimentary canal (midgut and hindgut). Bacterial colonies exhibiting different morphologies with different growth rates were observed after 12 h–48 h incubation (Fig. 1A). Six of these colonies were randomly chosen and maintained in the laboratory for all further experiments. In order to identify the cultured bacterial species, sequences from their small subunit ribosomal RNA (16S rRNA) genes were obtained and blasted against NCBI database (Fig. 1B).

**Figure 2:**
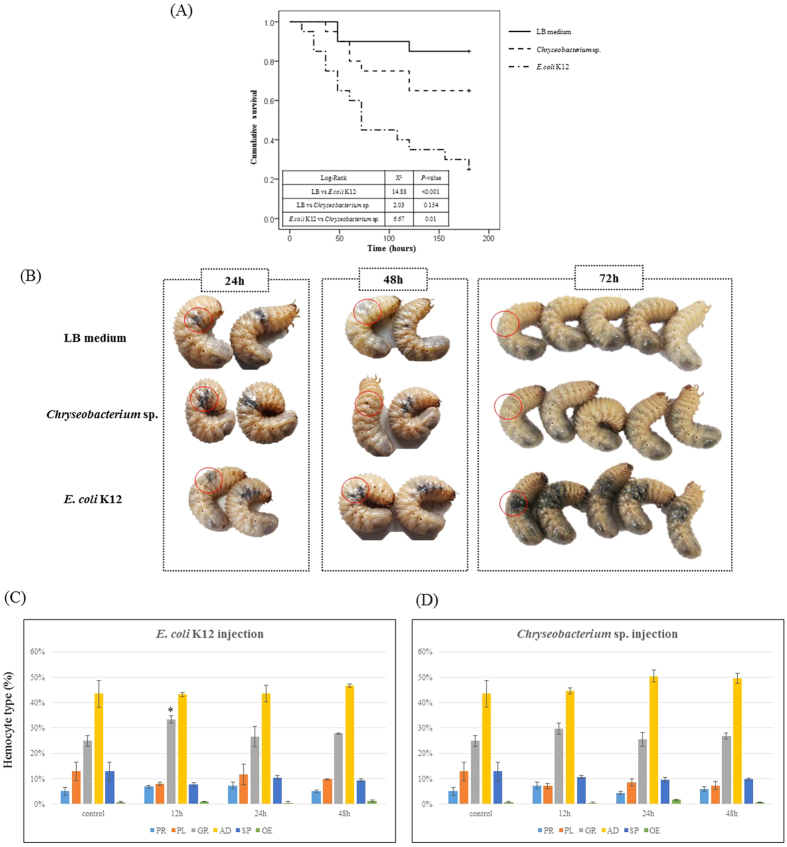
Larval survival and changes in hemocyte number in response to infection by *E. coli* K12 or *Chryseobacterium* sp. (**A**) Kaplan-Meier survival curve with log-rank test comparing survival of *Protaetia brevitarsis seulensis* larva infected with LB medium, *Chryseobacterium* sp., or *E. coli* K12. A *p*-value of less than 0.05 was considered statistically significant. Pairwise comparison: LB medium vs. *E. coli* K12, *p* < 0.001; LB medium vs. *Chryseobacterium* sp., *p* = 0.154; *Chryseobacterium* sp. vs. *E. coli* K12, *p* = 0.01. (**B**) Melanization in response to LB medium, *Chryseobacterium* sp., or *E. coli* K12 at 24 h, 48 h, or 72 h post-infection. The dark melanized spots gradually and completely disappeared at around 72 h post-infection with *Chryseobacterium* sp. or LB medium, but persisted in larvae infected with *E. coli* K12. Melanization within the injected area (marked by a red circle) was evident in *E. coli* K12-challenged larvae, but not in *Chryseobacterium* sp.- or LB medium-challenged larvae. (**C**,**D**) Five larvae per group were infected with *Chryseobacterium* sp. or *E. coli* K12 and the percentage of each of six circulating hemocyte types were assessed at different time points (12 h, 24 h, or 48 h). PR, prohemocytes; PL, plasmatocytes; GR, granulocytes; SP, spherulocytes; OE, oenocytoids; and AD, adipohemocytes. Results are expressed as the mean and standard deviation. (*P < 0.05). Challenged larvae were infected with *E. coli* K12 (**C**) or *Chryseobacterium* sp. (**D**).

**Figure 3:**
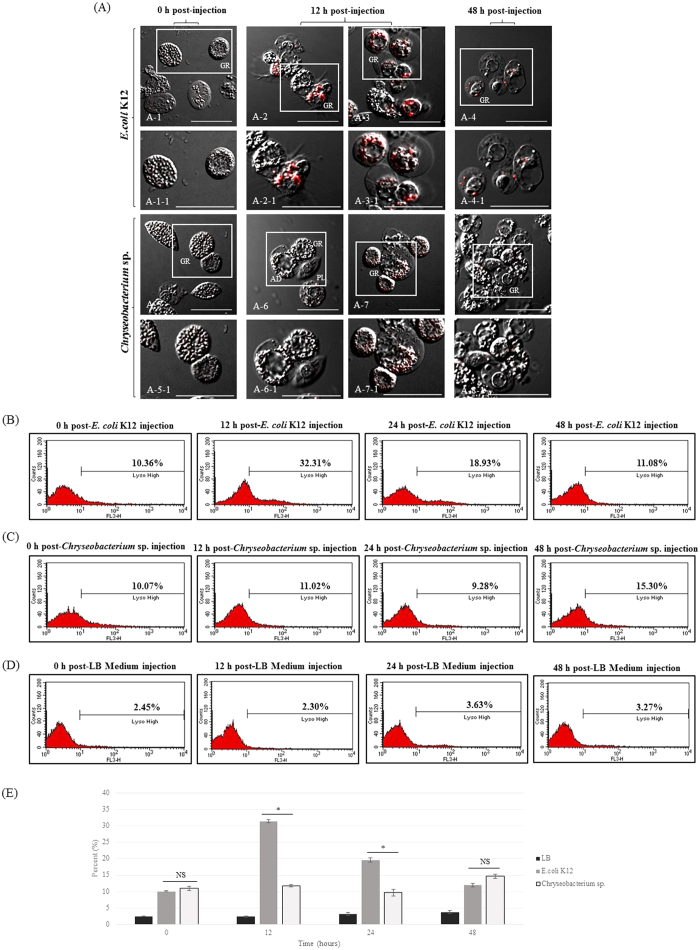
LysoTracker Red labeling of lysosomes in granulocytes and flow cytometric analysis after infection with *E. coli* K12 or *Chryseobacterium* sp. (**A**) Development of lysosomes after infection. Confocal fluorescent microscope images of granulocytes stained with LysoTracker Red (a lysosomal marker). *E. coli* K12 infection: (A-1) 0 h post-infection; (A-2, A-3) 12 h post-infection; (A-4) 48 h post-infection. *Chryseobacterium* sp. infection: (A-5) 0 h post-infection; (A-6 and A-7) 12 h post-infection; (A-8) 48 h post-infection. A-1-1, A-2-1, A-3-1, A-4-1, A-5-1, A-6-1, A-7-1, and A-8-1 are higher magnification images of the regions shown in the insets in panels A-1 through to A-8. GR, granulocytes; PL, plasmatocytes. Scale bar = 20 μm. At 12 h post-infection with *E. coli* K12, over 90% of granulocytes were strongly stained by LysoTracker Red (red in A-2 and A-3); however, staining was very faint after infection by *Chryseobacterium* sp. (A-6 and A-7). (**B**,**C**) Flow cytometry analysis of the total hemocyte population at 0~48 h post-infection with *E. coli* K12 or *Chryseobacterium* sp. At 12 h post-infection with *E. coli* K12, the percentage of stained granulocytes in the Lyso^high^ region increased from 10.36% to 32.31%. This gradually fell to 11.08% at 48 h post-infection. However, there was no increase in the population of stained granulocytes from *Chryseobacterium* sp.-challenged larvae (10.07% at 0 h, 11.02% at 12 h, and etc.). (**D**) Low levels of pathogen-associated lysosome activity were always observed in LB medium-challenged larvae (<5%). (**E**) Student’s *t*-test analysis of flow cytometry results to compare differences between *E. coli* K12- and *Chryseobacterium* sp.-challenged group. The experiment was repeated for three times. Error bars indicate Mean±SEM. *P < 0.05 (*t*-test). NS, not significant.

**Figure 4:**
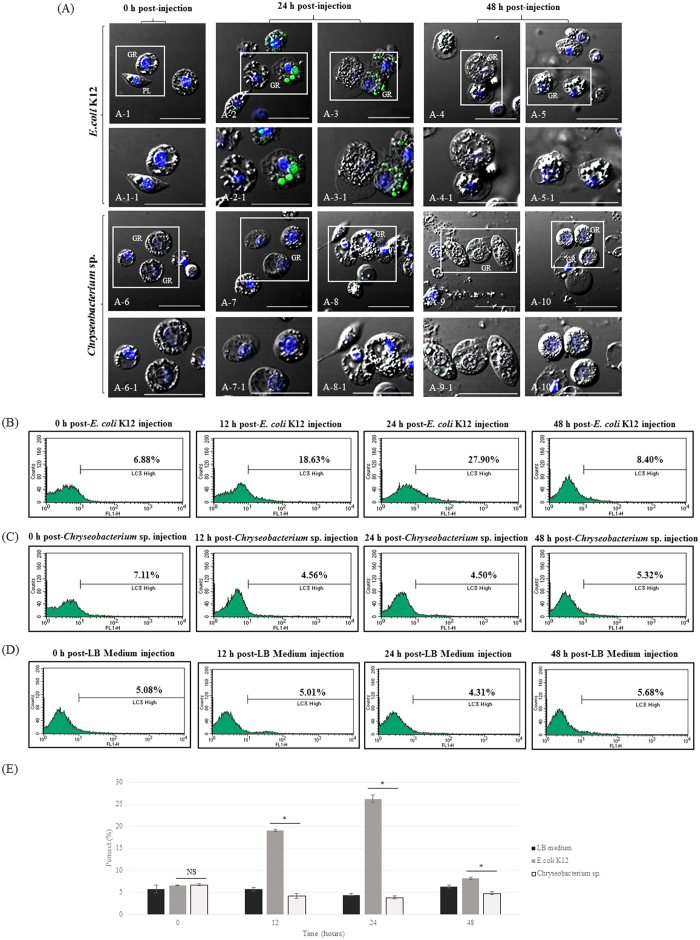
Green fluorescent-LC3 staining in granulocytes and flow cytometric analysis after infection with *E. coli* K12 or *Chryseobacterium* sp. (**A**) Formation of pathogen-related autophagosomes. Confocal fluorescent microscope images of granulocytes stained with DAPI (nuclei) and green fluorescent LC3 (autophagosomes). *E. coli* K12 infection: (A-1) 0 h post-infection; (A-2 and A-3) 24 h post-infection; (A-4 and A-5) 48 h post-infection. *Chryseobacterium* sp. infection: (A-6) 0 h post-infection; (A-7 and A-8) 24 h post-infection; (A-9 and A-10) 48 h post-infection. A-1-1 through to A-10-1 show the insets in panels A-1 through to A-10 at higher magnification. GR, granulocytes; PL, plasmatocytes. Scale bar = 20 μm. Many granulocytes were strongly stained by green fluorescent LC3 at 24 h post-*E. coli* K12 infection (A-2 and A-3), but staining was very faint in granulocytes after *Chryseobacterium* sp. infection (A-7 and A-8). (**B**,**C**) Total hemocytes at 0 ~48 h post-infection with *E. coli* K12 or *Chryseobacterium* sp. At 24 h post-infection with *E. coli* K12, the percentage of stained hemocytes in the LC3^high^ region increased from 6.88% to 27.90%, before falling again to 8.40% at 48 h post-infection. However, there were no observable changes in green fluorescence intensity in hemocytes from *Chryseobacterium* sp.-challenged larvae (7.11% at 0 h and 4.50% at 24 h). (**D**) Low levels of pathogen-associated autophagosome activity were always observed in LB medium-challenged larvae (<7%). (E) Student’s *t*-test of flow cytometry results to compare differences between *E. coli* K12- and *Chryseobacterium* sp.-challenged group. The experiment was repeated for three times. Error bars indicate Mean±SEM. *P < 0.05 (*t*-test). NS, not significant.

**Figure 5:**
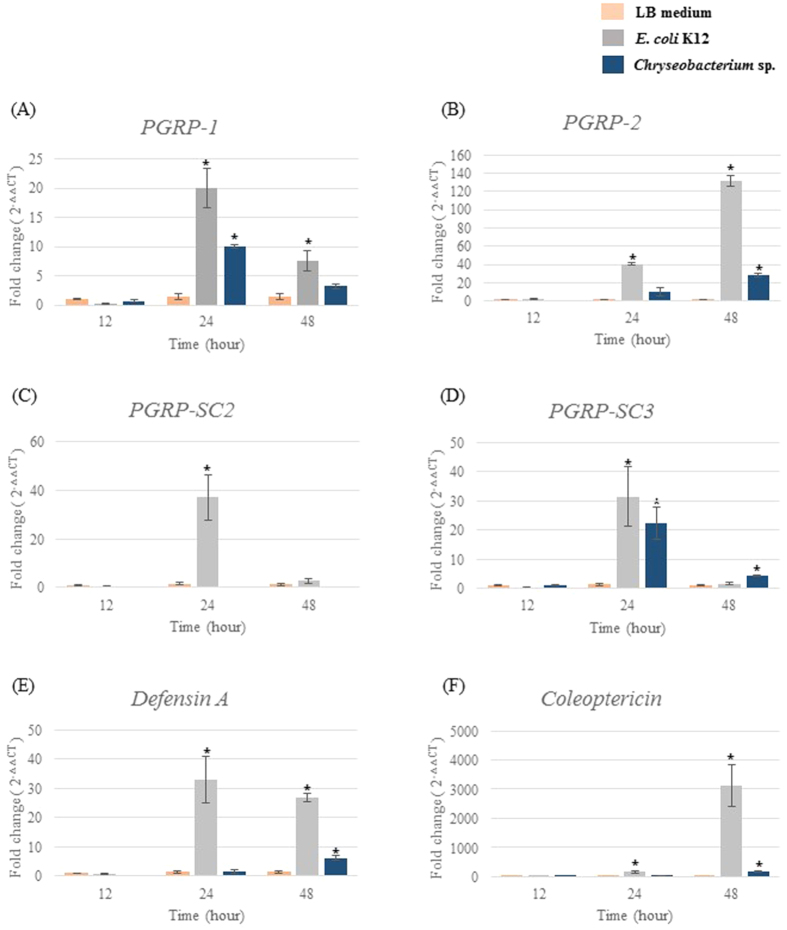
Expression of host immune genes at different time points after infection with *E. coli* K12 or *Chryseobacterium* sp. Expression of six immune-related genes [four peptidoglycan recognition proteins (**A**) *PGRP-1*; (**B**) *PGRP-2*; C, *PGRP-SC2*; and (**D**) *PGRP-SC3*) and two antimicrobial peptides (**E**) *defensin A* and (**F**) *coleoptericin*)] in fatbodies from LB-medium, *E. coli* K12, or *Chryseobacterium* sp.-challenged larvae. Expression was normalized to that in LB medium-challenged larvae. Values are expressed as the mean of three replicates ± SEM, each containing three larvae (nine larvae per condition). *P < 0.05 (*t*-test).

Next, to examine host innate cellular immune responses, the larval hemocoel was injected with *Chryseobacterium* sp., *B. subtilis, A. arilaitensis, B. amyloliquefaciens, L. xylanilyticus*, or *B. megaterium* (Fig. 1C,D). We then examined pathogenic-associated phagocytosis (including lysosomal formation and pathogen-related autophagosome vacuole formation) as an indicator of the cellular immune response against bacterial infection. As previously reported^14^, we found that a specific type of hemocyte (granulocytes) was associated with pathogenic lysosome formation and autophagy-related phagocytosis, which are efficient mechanisms for eliminating pathogens. Therefore, we used LysoTracker Red (a lysosome-selective stain) to identify acidified compartments in granulocytes, and green fluorescent staining-microtubule-associated protein 1A/1B-light chain 3 (LC3) to identify autophagosome formation (autophagosomes sequester materials intended for delivery to lysosomes). Granulocytes from bacteria-challenged larvae were stained with LysoTracker Red and green fluorescent-LC3 at 12 h post-infection and analyzed by flow cytometry. As shown in Fig. 1C,D, low levels of pathogen-associated lysosome- and autophagy-activity were observed in *Chryseobacterium* sp.-challenged larvae (LysoTracker Red-positive cells, 8.03% (C-1); green fluorescent-LC3-positive cells, 8.98% (D-1)). However, after infection with *L. xylanilyticus*, the percentage of cells showing pathogen-associated lysosome- and autophagy-activity increased to 38.84% and 42.01%, respectively (Fig. 1C-5,D-5). Infection with *B. subtilis, A. arilaitensis, B. amyloliquefaciens*, and *B. megaterium* led to moderate lysosome- and autophagosome-related immune responses (C-2; 18.95% and D-2; 19.60%, C-3; 14.19% and D-3; 30.42%, C-4; 12.25% and D-4; 11.52%, C-5; 38.84% and D-5; 42.01%, and C-6; 15.78% and D-6; 9.17% respectively).

To better understand the evolutionary relationship between *Chryseobacterium* sp. and other *Chryseobacterium* lineages, we next performed phylogenetic analyses using MEGA 6.0 software (Fig. 1E)^34^. Neighbor joining analysis and construction of a phylogenetic tree revealed that this bacterial strain closely related to the genus *Chryseobacterium*. Indeed, this bacterial strain showed 99.6% sequence identity with *Chryseobacterium* sp. IMER-A-2–17, 98.9% with *Chryseobacterium* sp. MH48, 98.6% with *Chryseobacterium* sp. IMER-A-2-9, and 98.3% with *Chryseobacterium* sp. AU939 (Fig. 1E).

### Larval survival and immune response to infection by *Chryseobacterium* sp. or *E. coli* K12

To investigate whether *Chryseobacterium* sp. is non-pathogenic in this insect, we analyzed host survival after infection with *Chryseobacterium* sp. After injection with either LB medium, *Chryseobacterium* sp., or *E. coli* K12, we then monitored survival every 12 h for 180 h (Fig. 2A). Larvae were injected with 10^6^ colony-forming units because this is the dose of *E. coli* K12 required to kill ~50% of larvae after 72 h. As shown in Fig. 2A, the survival rate of larvae injected with *E. coli* K12 was <50% at 72 h post-infection; however, 75% and 90% of larvae injected with *Chryseobacterium* sp. or LB medium survived (Fig. 2A). After 180 h, 65% of *Chryseobacterium* sp.-challenged larvae and 85% of LB medium-challenged larvae were alive as opposed to <30% of *E. coli* K12-injected larvae (Fig. 2A), respectively. There was better survival of *Chryseobacterium* sp.-challenged group compared that of the *E. coli* K12-challenged group (*p* = 0.01) but, there was no significant difference between LB medium- and *Chryseobacterium* sp.-challenged group (*p* = 0.154) (Fig. 2A). In addition, microscopic analysis revealed no immune-related morphological changes in granulocytes from *Chryseobacterium* sp.-challenged larvae. However, granulocytes from *E. coli* K12-challenged larvae showed amoeba-like, lobopodia-like, and fan-like structures, which are indicative of immune activation^4^.

Host melanization in response to pathogen infection is an important indicator of immune or wound healing responses in vertebrates and invertebrates. Therefore, we examined melanization in an area injected with either LB medium, *Chryseobacterium* sp., or *E. coli* K12. Melanization in response to *E. coli* K12 was initiated at 12 h post-infection and spread over the entire body by 24 h post-infection; this was maintained at 72 h post-infection (Fig. 2B). Melanization in response to *Chryseobacterium* sp. or LB medium also occurred up until 24 h post-injection, but this gradually disappeared and was completely absent at 72 h post-injection (Fig. 2B; injection site indicated by the red circle).

Changes in hemocyte number are also often used as an important indicator of innate cellular immune responses. Therefore, we calculated the total hemocyte count (THC) in larvae infected with either *E. coli* K12 or *Chryseobacterium* sp. (Fig. 2C,D). The percentage of granulocytes in *E. coli* K12-challenged larvae increased significantly (*P* = 0.01; *p < 0.05) at 12 h, before gradually decreasing at 48 h (Fig. 2C). However, the percentage of granulocytes in *Chryseobacterium* sp.-challenged larvae at 0 h or 24 h post-infection remained unchanged (Fig. 2D). The percentage of plasmatocytes, oenocytoids, spherulocytes, prohemocytes, and adipohemocytes was unchanged in both *E. coli* K12- and *Chryseobacterium* sp.-challenged larvae (*P* > 0.05; (t-test)) (Fig. 2C,D).

### Lysosome formation in granulocytes in response to infection by *Chryseobacterium* sp. or *E. coli* K12

To further explore whether the granulocytes from *Chryseobacterium* sp.-challenged larvae generated phagosomes or lysosomes in the cytoplasm, we stained granulocytes with LysoTracker Red and compared them with granulocytes from *E. coli* K12-challenged larvae (Fig. 3A). At 0 h post-injection of *E. coli* K12, the granulocytes contained no red fluorescent lysosomes (Fig. 3A-1). However, after 12 h, >90% of granulocytes showed strong staining with LysoTracker Red, particularly the highly polymorphic vacuoles (Fig. 3A-2,A-3). The red fluorescent signal gradually decreased up to 48 h post-injection (Fig. 3A-4). By contrast, most granulocytes from *Chryseobacterium* sp.-challenged larvae showed only faint staining with LysoTracker Red at 12 h and 48 h post-infection (Fig. 3A-6).

To quantify the red fluorescent signal in *E. coli* K12-, *Chryseobacterium* sp.-, or LB medium-challenged larvae, we stained hemocytes with LysoTracker Red and examined fluorescence at 0 h, 12 h, 24 h, or 48 h post-infection (Fig. 3B–D). Again, we detected red fluorescence in granulocytes from *E. coli* K12-challenged larvae at 12 h post-infection (Fig. 3B; 32.31% Lyso^high^). The signal then decreased gradually at 24 h and 48 h (18.93% and 11.08% Lyso^high^, respectively) (Fig. 3B). By contrast, there was no increase in the red fluorescent signal in granulocytes from *Chryseobacterium* sp.- or LB medium-challenged larvae at any time post-infection (Fig. 3C,D). As shown in Fig. 3E, compared to that of the *Chryseobacterium* sp.-challenged group, pathogen-associated lysosome activities in the *E. coli* K12-challenged group were significantly increased at 12 h or 24 h. These results reconfirmed the formation of phagosomal or lysosomal compartments within granulocytes from *E. coli* K12-challenged larvae; however, this was not the case in *Chryseobacterium* sp.- or LB medium-challenged larvae.

### Autophagosome formation in granulocytes in response to infection by *E. coli* K12 or *Chryseobacterium* sp

To determine whether autophagosomes were generated upon *Chryseobacterium* sp. infection, we examined *GFP-LC3* expression in hemocytes by fluorescence microscopy and flow cytometry. The accumulation of pathogen-related autophagosomes within granulocytes was examined by confocal microscopy (Fig. 4A). At 0 h after *E. coli* K12-injection, granulocytes did not emit a green fluorescent signal (LC3) (Fig. 4A-1). However, high levels of LC3 accumulated in granulocyte vacuoles at 24 h post-infection (Fig. 4A-2,A-3); the level decreased again at 48 h post-injection (Fig. 4A-4,A-5). By contrast, granulocytes from larvae injected with *Chryseobacterium* sp. were negative for LC3 at 0~48 h post-injection (Fig. 4A-6~A-10).

In flow cytometry analysis, at 24 h post-infection with *E. coli* K12, 27.90% of granulocytes emitted a fluorescent signal compared with 6.88% at 0 h (Fig. 4B). At 48 h post-injection, the LC3 signal decreased gradually (8.40% LC3^high^ cells). However, granulocytes from larvae infected by *Chryseobacterium* sp. or LB medium did not show increased emission of a fluorescent signal at any time point (Fig. 4C; 7.11~5.32% LC3^high^ and Fig. 4D). Compared to that of the *Chryseobacterium* sp.-challenged group, pathogen-associated autophagosome activities in the *E. coli* K12-challenged group were significantly increased at 12 h, 24 h, or 48 h (Fig. 4E).

### Expression of immune-related genes in *E. coli* K12- and *Chryseobacterium* sp.-challenged larvae

To examine the expression of host innate immune genes after infection with *Chryseobacterium* sp., we analyzed the expression profiles of a set of molecular pattern recognition proteins (which are usually activated upon interaction with microorganisms or microorganism-derived molecules) and *AMPs* (which are usually synthesized by the fat body in response to infection and secreted into the hemolymph). We previously isolated a number of *PGRP* orthologs and *AMPs* from *P. brevitarsis seulensis*, including *PGRP-1, 2, SC2, SC3, defensin A*, and *coleoptericin*^33^. Therefore, we used sequences derived from fragments of these genes (and the actin housekeeping gene) as reference genes for quantitative real-time polymerase chain reaction (qRT-PCR) analysis (Fig. 5A~F). Transcription of these genes was examined at 12, 24, and 48 h post-infection with LB-medium, *E. coli* K12, or *Chryseobacterium* sp. At 24 h post-infection with *E. coli* K12 or *Chryseobacterium* sp., *PGRP-1* expression was higher than after *LB-medium* infection (Fig. 5A). However, expression fell significantly at 48 h post-infection, whereas that in *E. coli* K12-challenged larvae remained high until 48 h post-infection (Fig. 5A). *PGRP-2* expression was also significantly higher than that after *LB-medium* infection at 48 h post-infection with *E. coli* K12 or *Chryseobacterium* sp. (Fig. 5B). As shown in Fig. 5C,D, expression of *PGRP-SC2* and -*SC3* was more than 30 times higher than in LB-medium challenged larvae at 24 h post-infection. Especially, that of *PGRP-SC2* was also more than 20 times higher than in *Chryseobacterium* sp.-challenged larvae at 24 h post-infection. Similarly, expression of *defensin A* increased by 30-fold in *E. coli* K12-challenged larvae at 24 h post-infection (Fig. 5E). In addition, expression of *coleoptericins* increased by more than 100-fold at 24 h post-infection and by more than 3,000-fold at 48 h post-infection with *E. coli* K12 (Fig. 5F). However, both *defensin A* (6-fold) and *coleoptericin* (182-fold) were slightly or moderately expressed in *Chryseobacterium* sp.-challenged group at 24 h or 48 h post-infection (Fig. 5E,F).

## Discussion

Generally, the lumen of the insect gut is densely colonized by various commensal or symbiotic microorganisms, which comprise <20~30 taxa according to 16S rRNA sequence analysis^35^. In addition, Yun *et al*.^25^ reported that the microbiota within the guts of various insects were dominated by *Proteobacteria, Firmicutes, Bacteroidetes, Actinobacteria, Tenericutes*, and unclassified bacteria^25^. Therefore, the lumen of the insect gut is a complex natural habitat occupied by various microorganisms, and there are multiple and complex interactions between them that enable them to survive. Thus, it is difficult to investigate the immunological relationships between gut-inhabiting microorganisms and host immunity.

To overcome these challenges, we cultured bacteria from the intestine of *Protaetia brevitarsis seulensis* and randomly selected or isolated six species of bacteria, which are probably gut-inhabiting microorganisms. Among these, we identified *Chryseobacterium* sp., which is probably a commensal/symbiont adapted to life in the gut of various insects^36^,^37^,^38^,^39^. *Chryseobacterium* sp. is a Gram-negative, aerobic, non-motile, yellow-pigmented, rod-shaped bacterium that belongs to the family *Flavobacteriaceae*; most members of this family are found in soil and in fresh or seawater habitats^40^. *Chryseobacterium* was originally isolated by Vandamme *et al*.^41^; since then, many species have been identified in soil, clinical samples, and the guts of insects^42^,^43^. Recently, *Chryseobacterium* sp. was identified in the gut of the wood-boring beetle *Anoplophora chinensis*, in which it has the ability to degrade cellulose and/or aromatic compounds^38^.

As shown in Fig. 1C, we found that five species of bacteria (not including *Chryseobacterium* sp.) moderately or strongly activated pathogen-associated lysosomal and pathogen-related autophagosome responses. This raised the question of why *Chryseobacterium* sp. alone induces only a weak immune response compared to other bacteria. Gut-inhabiting microorganisms can interact with host immune cells in different ways, and their survival in the host is dependent on how well adapted they are to the host^44^,^45^. As shown in Figs 3 and 4, most granulocytes from *Chryseobacterium* sp.-challenged larvae showed weaker lysosomal or autophagosome activity than that of *E. coli* K12 used as the infective agent. Activated granulocytes contain phagosomes, lysosomes, and autophagosomes, all of which clear pathogens and engulf potentially hazardous substances^4^. These immune-related morphological changes are also observed in phagocytes from other insects such as flies, mosquitoes, and beetles, although the type of professional phagocyte differs according to the insect species^3^,^5^,^12^. Macrophages or phagocytes in vertebrates and invertebrates are the front line of immune defense against virulent pathogens, and recognition of invading pathogens by these cells is the key step in generating immune responses^46^,^47^. Macrophages or phagocytes engulf potentially hazardous substances, a process that depends on the morphological shape of the pathogen or on expression of certain molecules on the cell membrane^4^,^12^. To rule out the possibility of a weak cellular immune response induced by *Chryseobacterium* sp. because their morphological characteristics were different from those of other microorganisms, we analyzed *E. coli* K12 and the *Chryseobacterium* sp. under a scanning electron microscope (SEM) (Fig. supplement 1A,B). We found that the shape and size of *Chryseobacterium* sp. were very similar to those of *E. coli* K12: a typical rod-shape of approximately 2 μm in length. Therefore, we concluded that morphological differences were unlikely to affect the ability of granulocytes to engulf the bacteria.

*Chryseobacterium* sp. may elicit a considerably low immune reaction against granulocytes by altering expression of pathogen-associated molecular patterns (PAMPs) such as outer membrane protein (OmpA), peptidoglycan, LPS, lipoproteins, β-1,3-glucan, bacterial flagellin, and various porins, all of which are key molecules detected by professional phagocytes. This down regulation of PAMPs is common to many bacteria^48^. Weiss *et al*.^23^ demonstrated that OmpA expressed by virulent bacteria is a strong inducer of host immunity. Thus, it appears that differences between avirulent and virulent pathogens are due to differences in the expression of common membrane constituents^23^,^49^. Recently, Kim *et al*.^50^ showed that the absence of the bacterial lipopolysaccharide O antigen in the cell envelope was responsible for the increased susceptibility of the gut inhabiting bacteria to host antimicrobial peptides^50^. PAMPs are recognized by a group of proteins known as PRRs (pattern recognition receptors), which include βGRPs, GNBPs, C-type lectins, scavenger receptors, and PGRPs, which are generally classified as short (S) or long (L) forms^51^,^52^,^53^,^54^. We recently reported that *P. brevitarsis seulensis* expresses orthologs of *PGRP-1, -2, -3, -LB, -LC, -LE, -SC2,* and *-SC3* 33. Bacterial motility is also essential for the successful symbiosis between host insect and gut inhabiting bacteria^55^,^56^.

As shown in Fig. 2A,B, larvae injected with *E. coli* K12 increased melanin production, which reduced their survival after infection. However, although larvae injected with *Chryseobacterium* sp. or LB medium did show slightly elevated levels of melanization at 24 h post-injection, this gradually and completely disappeared by 72 h (Fig. 2B). Melanization generally plays a role in thermoregulation, UV resistance, tolerance to desiccation, and resistance to abrasion^57^. Melanization in the cuticle is also another powerful immune mechanism that produces melanin at the site of wounds and provides resistance to infection; this type of melanization is visible as dark brown deposits^58^. Here, we found that expression of *PGRP-1* transcripts increased by more than 10 times at 24 h post-infection with *Chryseobacterium* sp. before decreasing at 48 h post-infection. These results were also coincident with melanization in Fig. 2B, which almost completely disappeared after about 72 h post-infection with *Chryseobacterium* sp. or LB medium. By contrast, we observed a continuous increase in *E. coli* K12-challenged larvae for up to 48 h (Fig. 5A). Although the level of *PGRP-1* transcripts need to be examined for more than 48 h, it seems likely that an increase of *PGRP-1* transcripts might be maintained since melanization induced by infection with *E. coli* K12 remained high at 72 h (Fig. 2B). We also observed that *PGRP-2* expression was kept constant higher during *E. coli* K12 infection (Fig. 5B). PGRP-1 and 2 activate PO (melanization) in flies, silk moths, and beetles^53^,^54^,^59^. In particular, Hd-PGRP-1 from *Holotrichia diomphalia (Korean black chafer) recognizes* β-1,3-glucan and induces prophenoloxidase (PPO) activity via serine proteases, which are clip domain enzymes that generate phenoloxidase (PO)^55^,^60^. In addition, we previously showed that melanotic encapsulation of pathogens mainly occurs in granulocytes activated only after infection with *E. coli* K12 4. Therefore, PO-related melanization is adhesive elements to pathogen surface including hemocyte surfaces^61^.

As shown in Fig. 5C, expression of *PGRP-SC2* increased 30-fold at 24 h post-infection with *E. coli* K12; however, expression of *PGRP-SC2* was very low in *Chryseobacterium* sp.-challenged larvae. Although further confirmation is required, the expression of *PGRP-SC2* could possibly be linked with bactericidal activity. *PGRP-SC2* and *-SC3* from this insect were more than 90% identical with *PGRP-S2 and PGRP-S3* from *Anopheles gambiae; both PGRP-S2 and PGRP-S3 are known as secreted* bactericidal *amidases*^16^. Hence, these results suggest that *Chryseobacterium* sp., which does not induce *PGRP-SC2* expression, is non-pathogenic or well adapted in this insect.

Finally, we examined expression of *AMP*s in response to *E. coli* K12 and *Chryseobacterium* sp. infection. AMPs are the last proteins to be secreted by the innate immune pathway. These proteins directly bind and kill bacteria in insects. Defencin A and coleoptericin act as AMPs in this insect^33^,^62^ and their expression is indicative of an AMP response. As shown Fig. 5E,F, *defensin* transcripts were upregulated in *E. coli* K12-challenged larvae (by more than 30-fold). In addition, expression of *coleoptericin*-like transcripts increased in *E. coli* K12-challenged larvae (by more than 3,000-fold), but were expressed weakly or moderately in *Chryseobacterium* sp.-challenged larvae. Anselme *et al*.^63^ showed that several immune genes, including *coleoptericin*, are highly overexpressed in the bacteriome, which is a specialized organelle that hosts endosymbiotic bacteria in some insects^63^. This AMP-driven selective pressure on endosymbiotic bacteria might be required to maintain the optimum number of microorganisms in the insect gut. Therefore, the very weak or moderate expression of *defensin* and *coleoptericin* transcripts in *Chryseobacterium* sp.-challenged larvae is probably due to the lack of a strong immune response, unlike the response to *E. coli* K12.

In summary, we cultured gut-inhabiting bacteria *in vitro* and examined host cellular and humoral immune responses against the bacteria. Compared to that of the *E. coli* K12-challenged larvae, we observed weaker or more moderate immune activities (e.g. lysosome and autophagosome formation in granulocytes or expression of immune-related genes) in *Chryseobacterium* sp.-challenged larvae. These results suggested that *Chryseobacterium* sp. may not have strong effector(s) or well-adapted to host immune system. It is very difficult to study the host immunological relationship between single bacterial taxa in the gut due to complex interactions between microorganisms that enable them to survive. The strategies used in this study can contribute to the ongoing investigation of the interactions between host immune system and single bacterial taxa inside of gut.

## Methods

### Insects, isolation and identification of intestine-adapted bacteria

The white-spotted flower chafers, *Protaetia brevitarsis seulensis* (Coleoptera: Cetoniidae) were reared and maintained as previously described^64^. In addition, the handling procedures of larvae including sample preparation, infection, and image analysis were also performed as previously described^4^,^12^.

The bacterial isolation from the gut of insects was performed as previously described^65^. Briefly, 10 last instar larvae were sterilized for 10 seconds in 95% ethanol and rinsing with sterile distilled water prior to dissection. The whole gut was aseptically removed under a stereomicroscope using insect pins and placed in 1.5 ml micro centrifuge tube containing 0.5 ml of phosphate buffer saline (NaH_2_PO_4_, Na_2_HPO_4_ and NaCl (pH = 7.2)). The dissected gut were squeezed several times using glass pestle, 1 ml of PBS was added, and 3,000 rpm at 4 °C for 15 seconds to separate the microorganisms from the gut wall. Ten-fold dilution of the bacterial suspension was filtered in 0.22 μm filter and the dilutions of the gut suspension were cultured immediately on SOC starch agar plates (2% tryptone, 0.5% Yeast extract, 8.56 mM NaCl, 2.5 mM KCl, _dd_H_2_O to 1000 mL, 10 mM MgCl_2_, 10 mM MgSO_4_, and 30 mM glucose). Then, six selected bacterial species including *E. coli* K12 were grown in LB media (although they grew more slowly) for 12 to 24 h at 28 °C and 0.5 ml liquid bacterial isolates were stored in liquid media containing 20% glycerol in cryovials at −80 °C for further processing. All the isolated colonies were observed under microscope to check their colony morphologies i.e. color, shape, and pigmentation. Bacterial colonies were randomly selected and singularly plated on new agar plate. For 16S rRNA gene analysis of gut-inhabiting bacteria, the genomic DNA extracts from each bacteria. Next, PCRs were performed to amplify 1.4 to 1.5 kb of the 16S rRNA gene from all the genomic DNA samples using 27F (5′-AGAGTTTGATCMTGGCTCAG-3′) and 1492R (5′- TACGGYTACCTTGTTACGACTT-3′). The PCR conditions comprised: 3 minutes at 94 °C, followed by 30 cycles of 94 °C for 30 seconds, 58 to 48 °C for 30 seconds (the temperature was decreased by 1 °C every cycle for 10 cycles and then held at 48 °C for 20 cycles), 72 °C for 1 minute, followed by a final extension step at 72 °C for 20 minutes. The purified PCR products were sequenced at Macrogen, Korea, using 785F (5′-GGATTAGATACCCTGGTA-3′) and 907R (5′-CCGTCAATTCMTTTRAGTTT-3′). We obtained 16S rRNA sequences data (1.47 kb, 1.51 kb, 1.48 kb, 1.67 kb, 1.58 kb, and 1.49 kb for *Chryseobacterium* sp. (KX371567), *Bacillus subtilis* (KX369580), *Arthrobacter arilaitensis* (KX369581), *Bacillus amyloliquefaciens* (KX369577), *Bacillus megaterium* (KX369578), and *Lysinibacillus xylanilyticus* (KX371346).

### Bacterial injection, hemocyte collection and counting, and statistical analysis

For injection, the last instar larvae were cold-anesthetized and a finely pulled glass needle was shallowly inserted into the dorsal vessel; around 40 μl (with 10^6^ colony-forming unit (CFU)) of cultured bacteria were injected into the hemocoel. For survival curve, the hemocoel of 60 larvae was injected and the larvae were divided into three groups: a *Chryseobacterium* sp.-injected group, an *E. coli* K12-injected group (a well-known pathogenic bacterium in this insect) [positive control], and an LB medium-injected group [negative control]. Hemocytes and fatbodies were collected and analyzed by direct puncture of the dorsal blood vessel or following dissection of larva abdominal cuticles of each larva at 0 h, 12 h, 24 h, and 48 h post injection to evaluate hemocyte morphology and the expression of immune-related genes. About 0.3 ml of hemolymph was collected in 1.5 ml micro centrifuge tube containing anti-coagulant solution (98mM NaOH, 186mM NaCl, 17 mM EDTA, and 41 mM citric acid, (pH 4.5)). After centrifugation (1,000 g for 10 min at 4 °C), the plasma were removed. For the Total Hemocyte Counts (THC), hemocytes were placed in a sterile disposable hemocytometer slide (Ncubauer Improve, iNCYTO C-Chip DHC-N01. www.incyto.com) (10 μl capacity). The THC were counted in four squares using a light microscope and the percentages were determined per individual larva (Leica DMI 3000B; 40 × objective). The THC was calculated at 0, 12, 24, and 48 h post-infection. Statistical tests were performed using the Minitab software package. Significant difference of means was calculated using Student’s two-tailed t-test or one-way ANOVA at a probability (*P*) value of less than 5%. The 16S rRNA gene sequences were compared with the National Center for Biotechnology Information Genbank database using the BLASTn (http://www.ncbi.nlm.nih.gov/BLAST/) and the Ribosomal Database Project II (RDP II) (http://rdp.cme.msu.edu). A phylogenetic study was performed by program MEGA 6.0 and a phylogenetic tree was constructed with the computer package ClustalW and the relationships between isolates were determined by the neighbor joining method^66^,^67^. A bootstrap analysis was performed to evaluate the topology of the phylogenetic tree (Bootstrap = 1,000).

### Hemocytes staining, fluorescence-activated cell sorting (FACS) analysis, and survival analysis

Hemocyte labeling, FACS, and visualization including image processing and microscopy analysis in this insect were performed as previously described^4^,^12^. Briefly, hemocyte morphology and phagocytic activities including lysosomal formation and pathogenic related autophagosome were observed with an Olympus FV1000 confocal microscope and Olympus image application software. Especially, DAPI (4′-6-diamidino-2phenylindole; 5 μg/ml) for nucleus, fluorescently-conjugated phalloidin (F-actin cytoskeleton) (6.6μM; Molecular probes), LysoTracker Red (7.5 nM; Molecular Probes) for lysosomes, and a Cyto-ID Autophagy Detection Kit (Enzo) for autophagy protein, LC3 were used. Hemocytes were stained with each dye for 30 min, washed three times with PBS, fixed with 4% paraformaldehyde for 15 min, washed again three times with PBS, and mounted. 10,000 hemocytes per sample (three larvae) was determined by flow cytometry (a BD FACSCanto flow cytometer, BD Bioscience; San Jose, CA) and sample analysis was performed according to protocols developed for this application using FACSDiva software from BD Biosciences. The channels for green fluorescence and for red fluorescence were FL1 (530/30 band-pass) and FL3 (610/20 band-pass). The survival analysis was replicated on three consecutive days. On each day 10 larvae of each group were hemocoelic injected by LB medium, *Chryseobacterium* sp., or *E. coli* K12. Mortality of each group (LB medium, *Chryseobacterium* sp., or *E. coli* K12 injection group) were analyzed every 12 h until 180 h following injection. Kaplan Meier survival estimates and log rank test for comparison were used for time to death analysis for LB medium-, *Chryseobacterium* sp.-, or *E. coli* K12-challenged group. A significant *P* value from the log rank test was set at <0.05. Data were analyzed using IBM SPSS statistics 22.0.

### Quantitative real-time RT-PCR

To investigate the expression levels of immune-related genes, total RNA was extracted from each larva fatbodies at 12, 24, and 48 h post injection (Promega Corp., Madison, WI, USA) and first-strand cDNA was synthesized using SuperScript III First-Strand Synthesis SuperMix (Invitrogen, Karlsruhe, Germany). For real-time quantitative RT-PCR (qPCR), reactions were performed using 72-well Rotor with in the Rotor-Gene-Q (Qiagen, USA) with 5 μl SYBR GreenI master mix (Elpis-biotech, Korea), 0.2 μl of cDNA, and 0.4 μM each of gene-specific primers (Table supplement 1). The qPCR reactions were done in triplicate (40 cycles of 95 °C for 10 sec, 60 °C for 10 sec and 72 °C for 10 sec with a single 470 nm green channel fluorescence measurement). Expression was normalized to *β-actin* and calibrator (control group) was LB medium-challenged larvae. This replicate was assigned a relative concentration of 1. The statistical analyses of the qPCR data were performed using Prism 5.0 (GraphPad Software, San Diego, CA, USA). Significant difference of means was calculated using paired t-test (*p < 0.05).

## Additional Information

**How to cite this article**: Lee, J. *et al*. Immune tolerance to an intestine-adapted bacteria, *Chryseobacterium* sp., injected into the hemocoel of *Protaetia brevitarsis seulensis. Sci. Rep.* **6**, 31722; doi: 10.1038/srep31722 (2016).
